# The Influence of Suprarenals in Pneumonia

**Published:** 1902-10

**Authors:** 


### Abstracts and Selections.

### The Influence of Suprarenals in Pneumonia.

Ethan Allen Gray, in *New York Medical Record,* cites seven cases, covering a wide variety of pulmonary symptoms and complications, in which suprarenal capsule was used with invariably good results, high temperature was reduced, expectoration rendered more easy and freed from blood, while a steady and progressive improvement was seen in all the cases. C. R. M.

